# Massive penoscrotal haematoma following inguinal hernia repair: a case report

**DOI:** 10.1186/1752-1947-2-357

**Published:** 2008-11-21

**Authors:** Dharmendra K Shah, Jayesh Sagar

**Affiliations:** 1 Department of Surgery, S.S.G. Hospital and Medical College, Vadodara, Gujarat, 390001, India; 2 Division of Surgery and Interventional Science, Royal Free and University College Medical School, 9th Floor, Hampstead Campus, London, NW3 2QG, UK

## Abstract

**Introduction:**

Inguinal hernia is one of the commonest surgical conditions that one comes across in a surgical career. Operative repair is the only successful treatment for hernias. As with other surgical procedures, this is also associated with possible complications. Scrotal haematoma is one of the well-known complications following hernia repair, but massive penoscrotal haematoma requiring surgical intervention is very rare.

**Case presentation:**

A 53-year-old black man had undergone elective hernia repair. He underwent standard open hernia repair with a prolene mesh and developed massive scrotal haematoma which required drainage. Eventually he recovered well, although slowly.

**Conclusion:**

To achieve adequate bleeding control during and at the end of operation is the key preventive measure to avoid scrotal haematoma. Here, we report a case of massive penoscrotal haematoma following repair of a moderate sized inguinal hernia. We strongly emphasize the importance of adequate control of bleeding, even in small to moderate sized inguinal hernias in order to avoid such disastrous complications with long-term cosmetic disfigurement.

## Introduction

Inguinal hernia is the commonest of all of the hernias. Operative repair is the only acceptable method for treatment of inguinal hernias where possible. This is considered the most common operation and the operative procedure of choice for young training surgeons. As with any other surgical condition, hernia repair is also associated with different possible complications such as infection, bleeding, recurrence, scrotal swelling and nerve damage. These complications have been discussed in detail in the English literature. Penoscrotal haematoma, one of the complications, is a very well documented complication following inguinal hernia repair, however, massive penoscrotal haematoma requiring surgical intervention, is very rare and not yet reported in the English literature. Here, we present the case of a 53-year-old black man who developed massive penoscrotal haematoma following inguinal hernia repair that needed surgical exploration. Following inguinal hernia repair, scrotal haematoma or wound haematoma are treated conservatively. In rare occasions, they may need wound exploration or surgical drainage. Our patient developed a huge penoscrotal haematoma that required surgical drainage of the wound followed by drain placement. However, the patient did not respond to surgical treatment of the haematoma and had to undergo further drainage of the scrotal haematoma on the following day. As learned from this case report, we strongly emphasize meticulous surgical techniques to avoid and control bleeding during hernia repair. We also point out the importance of close observation in the recovery period and in the ward following such operations.

## Case presentation

**Figure 1:**
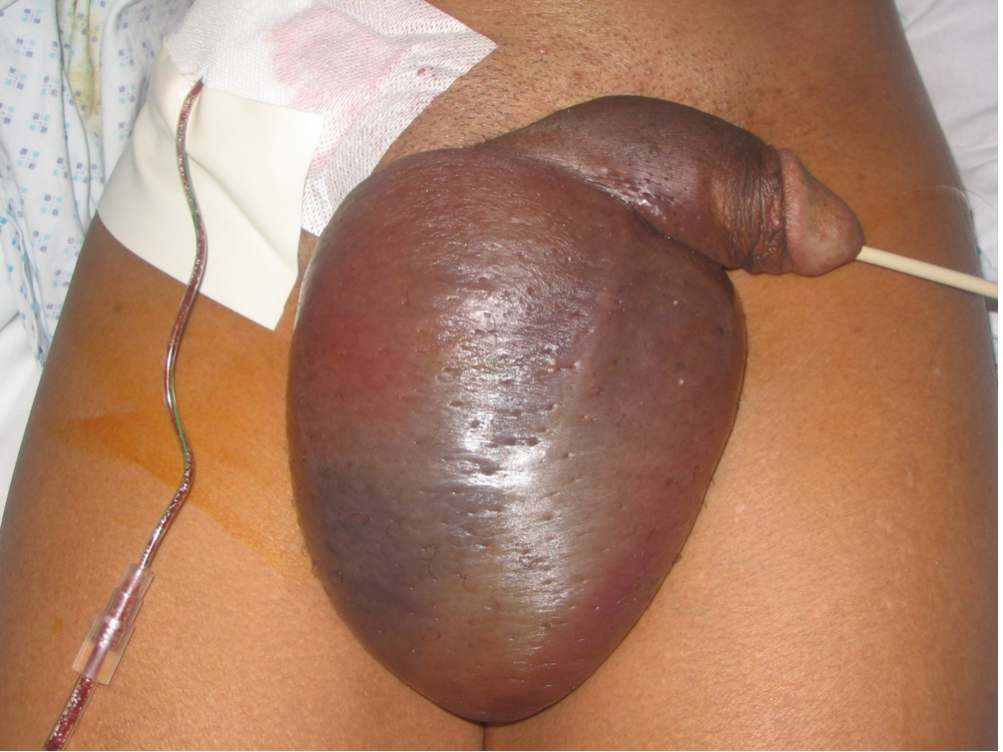
**Massive penoscrotal haematoma following inguinal hernia repair.** The drain is placed in the main wound following exploration of the right inguinal wound.

A 53-year-old black man had been admitted for elective right inguinal hernia repair and excision of a lipoma from his back at one of the private hospitals in London. He was fit and healthy without any medical problems. He had undergone open repair of a moderate-sized right inguinal hernia with a prolene mesh and excision of the lipoma from his back under general anaesthetic without any intra-operative complications. After the two hour operation, he complained of pain at the operative site. Examination of the local area revealed a massive penoscrotal haematoma. As he was stable haemodynamically, he underwent wound exploration under general anaesthesia. The bleeding spurts within the hernia wound were stopped and the wound was closed with a Radivac drain. A urethral catheter was inserted in the postoperative period as he could not pass urine. He was started on oral augmentin as prophylaxis. On the first postoperative day, his haemoglobin dropped to 7.1 gm% so he was given 2 units of blood. The drain had released about 20 ml of blood. On the second postoperative day, he was still in pain. Local examination revealed a massive penoscrotal haematoma (Figure 1) with no change in its size. He had further drainage of the scrotal haematoma under local anaesthesia and 200 ml of blood was evacuated. The wound was closed with a corrugated drain. The patient was investigated thoroughly by a haematologist and all blood investigations including coagulation profile, Factor XI bioassay, PFA-100 platelet function tests, vWF:antigen assay and vWF:collagen binding were normal. The drain was removed on the fourth postoperative day and the patient was discharged on augmentin. On a follow-up visit after 2 weeks, the patient was doing well without any complaints although with cosmetic disfigurement.

## Discussion

Inguinal hernia is one of the commonest surgical conditions affecting especially the male population. Surgical repair is the mainstay of treatment. As with any other surgical procedure, this is associated with possible complications. These include urinary retention, superficial wound haematoma, superficial wound infection, serous effusion, scrotal oedema, recurrence of hernia, persistent inguinal neuralgia, local hypoaesthesia, ischaemic orchitis and penoscrotal haematoma [1]. Most of these complications are of mild to moderate degree and can be treated by a conservative approach. Recent advances in different surgical techniques and equipment claim to have less complications but none are completely devoid of them [2].

Penoscrotal haematoma is one of the most common complications, and usually responds to a conservative approach in the form of rest and scrotal support. In doubtful cases, ultrasound evaluation of the penoscrotal haematoma/swelling is a useful guide to confirm the diagnosis [3] but in cases of massive haematoma, clinical diagnosis is obvious and does not necessarily require ultrasound. In cases of huge scrotal haematoma or unresolving haematoma, surgical drainage may be necessary. Massive penoscrotal haematoma is not uncommon in patients with bleeding disorders such as haemophilia where trivial trauma can trigger severe bleeding in the scrotum [4]. It has also been reported in patients following transfemoral cardiac catheterization [5], percutaneous transluminal angioplasty [6] and with rupture of the Dacron aorto-femoral graft [7]. It has also been reported as a complication following urological procedures [8,9]. Occurrence of penoscrotal haematoma following inguinal hernia repair is well documented in the medical literature but massive penoscrotal haematoma requiring surgical intervention is very rare. Although we agree that surgeons might have come across such complications more frequently, especially in the developing countries, this is the first time such a presentation has been reported in the English literature. As occurred in our patient, occasionally, such a massive haematoma may leave complications such as cosmetic disfigurement and unsatisfactory sexual performance. Usually scrotal haematoma following repair of an inguinal hernia involves discoloration of the scrotum and penis due to blood extravasation and haematoma of the scrotum without haematoma of the penis. As in our patient, the penile haematoma may be due to bleeding at the superficial dartos plane (the plane between the superficial dartos muscle of the scrotum and the dartos fascia of the penis).

The purpose of presenting this case report is to emphasize that meticulous and complete control of bleeding is important during hernia repair to avoid such complications. We strongly recommend adequate control of bleeding before closure of the wound in any kind of surgical procedure. Different techniques have been employed in practice such as the hitch-stitch and drain technique [10] to prevent bleeding and avoid significant postoperative haematoma. However, even simple techniques such as the use of diathermy and/or suture ligation of blood vessels along with meticulous surgical techniques may be helpful to avoid bleeding. In our patient, we could not determine why the surgeon failed to control bleeding before closure of the wound. This rule of prevention rather than cure does apply to all grades of surgeon, all grades of operation and in all sectors of health care. We also advise thorough investigations to rule out any bleeding tendency in patients with massive inguino-scrotal hernia so as to avoid any postoperative haematoma. This case also focuses on the importance of close observation of the patient by nursing staff in recovery as well as in the ward in the immediate postoperative period. This case report also raises a question over the possible use of a drain in the inguinal hernia. Although the use of a drain following repair of a large inguinal hernia is dubious and subjective, we prefer to use a drain in huge inguinal hernia repair and in doubtful haemostasis but we admit that our patient suffered from only a moderate sized inguinal hernia. One may argue that if we had used a drain in the first operation, we would have eliminated the need for a second operative intervention in this patient.

## Conclusion

We conclude that haemostasis is paramount in any surgical procedure irrespective of the nature of surgery. We also conclude that drains should be used in huge inguinal and inguino-scrotal hernias to avoid haematoma and scrotal swelling.

## Abbreviations

Factor XI: plasma thromboplastin antecedent; PFA: Platelet Function Analyzer; vWF: Von Willebrand Factor

## Consent

Written informed consent was obtained from the patient for publication of this case report and any accompanying images. A copy of the written consent is available for review by the Editor-in-Chief of this journal.

## Competing interests

The authors declare that they have no competing interests.

## Authors' contributions

JS was involved in the study concept and design, patient care, review of the literature and supervision of the work DKS contributed in the preparation of this manuscript along with the literature review.
